# Caregiver perspectives on patient capacities and institutional pathways to person centered forensic psychiatric care

**DOI:** 10.1371/journal.pone.0275205

**Published:** 2022-09-29

**Authors:** Leila El-Alti, Lars Sandman, Christian Munthe

**Affiliations:** 1 School of Health and Social Care, Edinburgh Napier University, Sighthill Court, Edinburgh, United Kingdom; 2 Department of Philosophy, Linguistics, and Theory of Science, University of Gothenburg, Gothenburg, Sweden; 3 Department of Medical and Health Sciences, Linköping University, Linköping, Sweden; University of Turku, FINLAND

## Abstract

The ethical discourse surrounding patients’ agential capacities, vis-à-vis their active participation in shared decision-making (SDM) in forensic psychiatric (FP) contexts, is an unexplored area of inquiry. The aim of this paper is to explore caregivers’ perceptions of patient agential capacities and institutional pathways and barriers to person centered care (PCC) in the context of FP. Following an exploratory qualitative design, we conducted eight semi-structured interviews with hands-on caregivers at an in-patient FP facility in Sweden. A deductive framework method of analysis was employed, and four themes emerged: “Fundamental Variability in Patient Capacity”, “Patient Participation: Narration or Compliance?”, “Antagonism Rooted in Power Struggles”, and “System Structure Thwarts Patient Release”. While the results generally paint a bleak picture for the possibility of a person-centered FP care, we describe a constrained version of PCC with high-level SDM dynamics which promotes a certain degree of patient empowerment while allowing care strategies, within set restrictions, to promote patient adherence and treatment progress.

## Introduction

We present a seminal attempt to assess how caregivers view the room for person centered care (PCC) approaches in forensic psychiatry (FP), in view of the peculiar circumstances of this type of care in relation to patient capacities and institutional complexity. The feasibility of calls to increase PCC, patient *participation*, or patient *collaboration* in FP depends on how caregivers view such prospects. This study not only highlights clearly perceived limitations in terms of patients’ agential capacities, but also how PCC in FP may be undermined by institutional factors vis-à-vis complex goals and partly conflicting ethos governing this type of care. On this basis, we discuss to what extent PCC-initiatives are desirable in FP, contingent on a deeper understanding of the ethical basis of FP and on which PCC goals are assumed.

## Background

FP institutionalization in Sweden typically requires being found guilty of a crime while under the influence of severe mental illness and accordingly sentenced by a court order to involuntary and indefinite FP care [1]. The notion of PCC in FP, as in other highly coercive inpatient psychiatric care, has attracted increased advocacy in recent years [2–5]. At the same time, serious doubt has been raised around what this would imply, and to what extent it is compatible with the nature of this type of care [6], particularly in relation to patient capacities and the coercive institutional context [4,7–9]. The notion of PCC is rather flexible, and need not assume one particular ethos or goal, so it remains open to analysis of the extent to which person-centered FP care is a viable notion. What is critical, however, is how caregivers, being the ones supposed to implement whatever idea of PCC is advocated, view the prospects. A recent study in Sweden suggests that there is no consistent way in which FP caregivers understand the concepts of PCC or patient *participation*, and that there is uncertainty about why these are advocated in the first place [10]. A similar result has also been generated for mental health care in general [3]. However, no study has so far looked at how forensic psychiatric staff view and reason with regard to the *feasibility* and *desirability* of different specific variants of PCC in relation to patient capacities and the institutional context of the care.

The notion and the goals of PCC are complex, but have recently been analyzed in general terms [11–13]. PCC is based on a holistic view of the patient as a complex person, whose own subjective narrative needs to be considered, besides the objective measurements of biomedically-defined parameters, and where healthcare professionals (HCPs) continuously employ shared decision-making (SDM) with the patient based on this narrative. The most common and ambitious objective of PCC is to empower and emancipate patients through a therapeutic alliance [13,14]. However, the point of PCC may also be less ambitious, as is illustrated by the many variants of SDM discussed in the literature, several of which seem to be aimed mainly at enhancing patient compliance with pre-defined care standards or prescriptions [15,16].

Sandman and Munthe have highlighted that the variants of PCC aiming for empowerment and enhancement typically assume a very ambitious notion of SDM–called *high-level dynamics*–where the patient and the HCP are assumed to be on equal footing regarding decision-making authority, and where the very goals of the care are open to deliberation and debate [15]. High-level dynamics imply strong assumptions about the patient’s agential capacities to participate in such SDM (in terms of collaboration, decision-making and responsibility), the power balance regarding authority, and a care context allowing for adjustments along whatever plans result from the SDM process. But in many cases these assumptions do not hold, making PCC in such contexts risky in terms of potentially violating professional ethics, disempowering patients, or outright harming them [12,17,18].

In the case of forensic and other highly coercive psychiatric care contexts, it is obvious that the assumptions about patient agential capacities can be questioned, since such capacities are often weakened by mental illness and thus constitute (one of) the main reason(s) why the patients have been committed to such care. Moreover, it is obvious that the coercive context motivated by considerations of general public safety is largely non-negotiable as an FP goal, and severely undermines assumptions about a power balance and the aim of patient emancipation [8]. At the same time, it has been observed that PCC and SDM may very well be employed to less ambitious ends in contexts where the main objective does not only concern the individual patient’s health or wellbeing, such as in public health measures [8]. However, it remains uncertain to what extent patient capacities for decision-making and responsibility can allow for any type of PCC in FP, and whether the institutional complexity of care (due to criminal/legal norms and general safety considerations) allow for capacities to be discharged constructively.

Agential capacities refer to ingredients in standard conceptions of decision-making capacity, namely understanding, appreciation, and reasoning [19], as well as capacity to take responsibility for made decisions and subsequent adherence or non-adherence to such decisions. Understanding and appreciation is a person’s ability to comprehend general and context specific information, while reasoning is the capacity to process and use this information, together with preferences, to make decisions. Capacity for responsibility for actions includes standard conditions of ability to comprehend the nature of the action, and the ability to control whether it is undertaken, as well as abilities to reflect morally on these actions, and adjust future behavior based on that [20]. The institutional complexity of FP includes its mix of aims from different sectors that may come into conflict: criminal law, healthcare, and general public safety [6,21].

Whichever form of PCC could be applied within FP care and to what extent it would be desirable to implement, depends on how caregivers providing this care to patients view the aforementioned factors of agential capacities and institutional complexity. In addition to the importance of involving caregivers in the ethical discourse which affects their practice, exploring caregivers’ views on the room for a desirable form of PCC in FP is vital for any implementation attempt. For instance, if caregivers see limitations to what forms of PCC might fit FP, implementation plans should be informed by this. Furthermore, such views of staff may also highlight aspects of FP care and structure which could be modified to facilitate pathways to a feasible and desirable PCC. Conversely, adopting a healthcare provision model without exploring how caregivers understand of the concept (and its associated ideas, processes, and values) or without assessing its contextual feasibility, risks becoming counterproductive or even harmful [1]. Mapping and analyzing the views of FP caring staff in these respects is thus crucial for the evaluation and design of implementation plans for PCC in FP.

## Aims and methods

### Aim

The aim of this study was to explore caregiver views and ideas regarding PCC, patient agential capacities, and institutional complexity at a high-security FP care facility. In particular, we wanted staff with hands-on patient care experience in FP to engage with ideas about what barriers and possible pathways there are for different forms of PCC in FP. Preliminary inquiries made clear that wards designated for care of patients in acute stages were not of interest, as the heavy restraints and medication of patients in these wards leave no room for PCC. This is not to imply that certain patients who are sedated or restrained (do not qualify for or) are undeserving of a PCC approach. However, restraining and heavily medicating patients due to an acute situation would temporarily but severely limit patient agential capacities and PCC processes (e.g., SDM), both of which are part of the research question. This does not only apply exclusively to FP but also extends to other healthcare settings, for instance, where patients are comatose due to an acute brain injury. For this reason, our study focused on wards which leave a varying degree of preliminary room for patient participatory care strategies.

### Methods

As mentioned earlier, as far as we know, no study has looked at feasibility of PCC in relation to patient agential capacities and contextual complexities in FP from the perspective of caregivers. Hence, we found it appropriate to choose an exploratory qualitative design. Since our aim was to elicit reasoning on specific theoretical topics and, at the same time, remain open for new theoretical categories and notions to be introduced regarding barriers and pathways for PCC, we opted for a framework analysis process as described by Ritchie and Spencer [22]. Our framework was based on preselected conceptions of PCC, agential capacities, and the institutional complexity of FP, to capture new ideas on barriers and pathways for different forms of PCC within FP. For this reason, following our preconceived framework, the interview guide mixed questions about broadly conceived agential capacities of patients and possible features of PCC in FP, with open-ended questions regarding barriers for PCC and pathways to overcome these. Another reason for choosing predefined categories was due to the researchers’ knowledge of philosophical and PCC literature, which would have inevitably influenced data collection and/or analysis had preconceptions and definitions not been explicitly specified [23].

**Fig 1:**
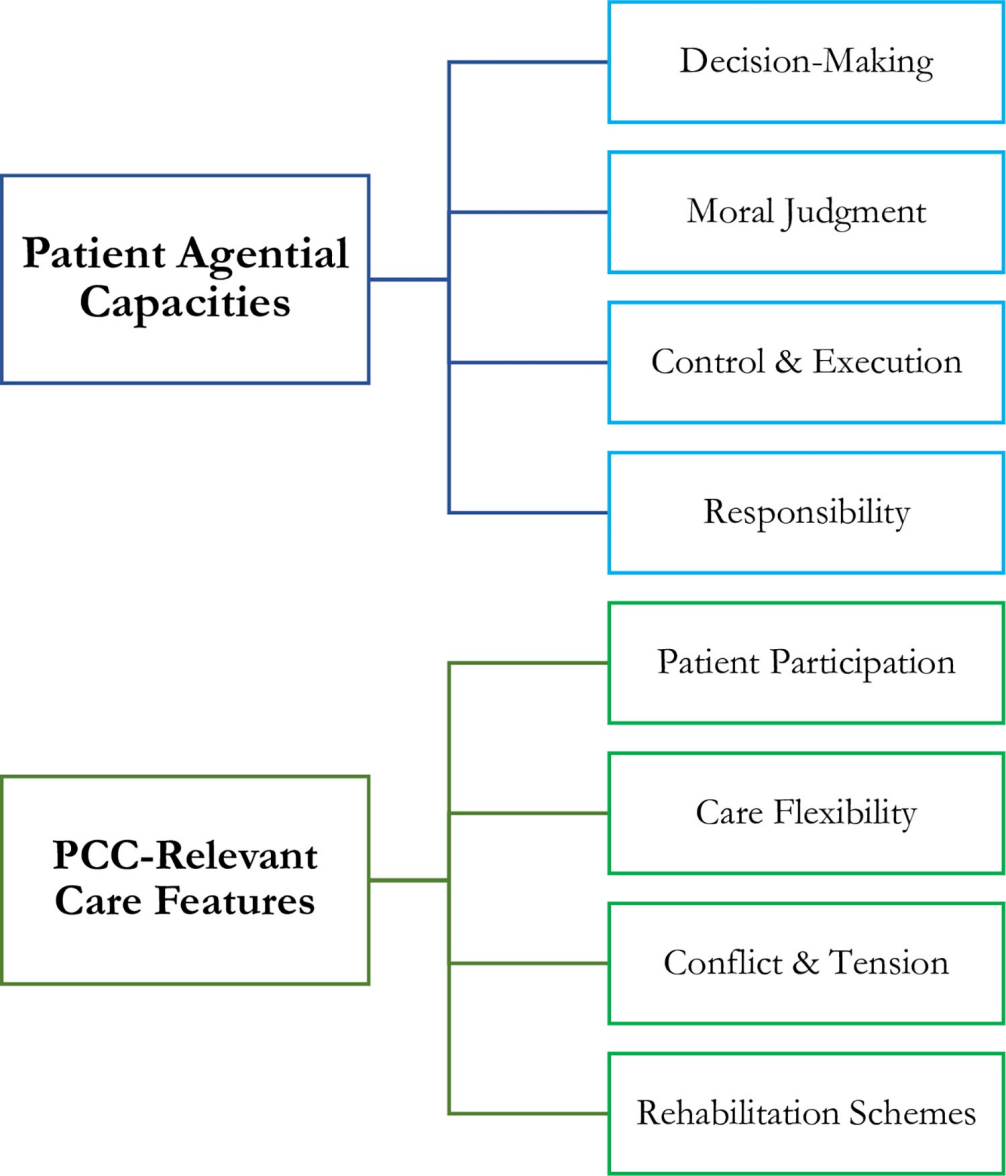
A tree diagram representing the categories of the analytic framework.

The selected categories of patient capacities (decision-making, control, moral judgments, responsibility) and PCC features (patient participation, care flexibility, conflicts, and rehabilitation strategies) were sketched in a tree diagram (Fig 1) and predefined (Table 1) to help produce the interview guide. Since the aim of this study was to elicit considered views and patterns of reasoning, rather than phenomenological lived experience, the interviews also contained elements of stimulating the subjects to explain, justify, and develop their views (see S1 and S2 Appendices).

**Table 1:** Definitions of framework categories, under two broad headings of patients’ agential capacities and care features of relevance to PCC.

**Patient** **Agential** **Capacities**	**Decision-Making**	Ability to determine suitable action(s) based on reasoning about relevant information in relation to one’s own views, wishes, goals, and/or values
	**Moral Judgment**	Ability to assess moral rightness or wrongness of own actions and/or of others’
	**Control & Execution**	Physical and/or mental ability to master emotions, impulses, and/or actions based on one’s judgment(s) or decision(s)
	**Responsibility**	Ability to commit oneself to executing actions on which one decided and/or to which one agreed, and accepting consequences of such actions
**PCC-Relevant Care Features**	**Patient Participation**	Active involvement of patient in decision-making, including (deliberating about) objective-setting, planning care, and sharing responsibility for executing plan of care
	**Care Flexibility**	Adaptability of care processes to accommodate for patient’s individuality, values, needs, desires, requests, and feedback
	**Conflict & Tension**	Discord of ethically relevant nature between caregiver(s) and patient, among caregivers themselves, or inside (a) patient group(s)
	**Rehabilitation Schemes**	Goals that care aims to achieve as well as strategies employed to influence or correct patient’s behavior(s) or action(s) toward these goals

### Recruitment

Participants were all recruited from suitable wards (see *Aim* above) at a forensic psychiatric facility in Sweden. This facility, like other FP facilities in Sweden, is entirely tax-funded, and publicly run by its respective county. The facility consists of (low, medium, and high security) inpatient and outpatient divisions and provides care to more than 200 patients at a time. The study was conducted in the in-patient division, which includes wards under highest security levels. We have chosen this facility in particular since access to potential participants has been made possible by key gatekeepers who have collaborated with the researchers on previous projects. As such, our sampling strategy fell under convenience sampling. It is important to note that these gatekeepers only facilitated contact with the facility’s director, who then made it possible to present the study at staff meetings and later interview potential participants inside the facility when they so preferred. This partly eased navigating the complex security protocols in order to gain access to (potential participants inside) the facility buildings, which would have otherwise been extremely difficult.

Information about the study was presented at staff meetings with the permission of the facility director, and all caregivers were invited to participate via a flyer distributed with the help of the clinic administration personnel. The invitation stressed that we primarily sought staff involved in the regular care of the patients, and included the researchers’ contact information so that caregivers can contact us themselves if they are interested in participation. Eight caregivers contacted the research team and expressed an interest to participate in the study, and all eight participants were subsequently included. Among our participants were three registered nurses, three treatment assistants, one nurse assistant, and one psychiatric nurse specialist; with educational backgrounds in nursing, psychiatry, psychology, social work, and treatment pedagogy. Participants cared for patients with a variety of psychiatric diagnoses including addiction disorder (6 out of 8) and/or personality disorders, psychosis, bipolar, and mixed diagnoses.

### Data collection

Date, time, and location of the interview were arranged to suit each participant’s preferences.

All interviews took place over several months in 2017, and were roughly one hour in length, audiotaped, and conducted in Swedish. Interview recordings were then transcribed verbatim and translated to English by a professional translation and transcription specialist. Data collection ceased when no further contact was made by potential participants following a reminder e-mail from the facility director.

### Data analysis

Our research falls at an interdisciplinary intersection compounded of *thick* concepts, such as moral agency, and complex models, such as PCC, which themselves lack a unified definition in their respective disciplines [13,24]. This made it practically impossible to phrase our interview questions to the participants without first identifying and defining some elements of moral agency as well as relevant aspects of what PCC in FP might amount to. Therefore, we opted for a *deductive* framework analysis wherein the framework categories were preselected and predefined, and could be explained to the participants if or when need arose. We believe that using ideas or concepts which participants are more likely understand and thus reason about, facilitated the formulation of our interview questions and the interview process.

However, because our interest lies in the caregivers’ thoughts and reflections regarding patient capacities and the care context, we also did not want to miss any findings in the data for which our analytic framework did not account. For that reason, instead of starting with the application of the framework to the data, we chose to proceed with open coding of each transcript followed by charting data summaries into a matrix, under the different framework categories [25]. Findings which did not fit under any of the categories were placed in a separate column labeled “Other”.

Hence, we made some adjustments to Ritchie and Spencer’s framework method of analysis, which states that data analysis proceeds through the following five stages: familiarization, identifying a thematic framework, indexing, charting, and finally, mapping and interpretation [22]. Since the categories of our framework were predetermined, we switched the first two steps in Ritchie and Spencer’s method [22] and replaced the indexing stage (which usually involves the application of the framework categories to the data) with open coding, followed by steps four and five of charting and interpretation, as described above.

Mixing deductive and inductive analysis allowed us to explore both our pre-specified notions of moral agency and the nature of PCC, as well as potentially unexpected findings in the data [25,26]. The themes, as presented in the results section, emerged through going back and forth over the data across different categories as well as across different participants. This process ended when no new themes appeared, and we felt that the themes portrayed the data sufficiently and accurately.

In order to enhance trustworthiness of the data analysis, investigator triangulation was employed. Data collection, transcription and translation, and data analysis were performed separately by three different people. To ensure confirmability, a detailed account of the data analysis steps was also shared by the researcher analyzing the data with the other two researchers, and was thoroughly discussed in multiple meetings spanning over several months. The Standards for Reporting Qualitative Research (SRQR) checklist was used as a guide for reporting in this paper.

### Ethics

Since the study could potentially process sensitive personal information of the participants, legally mandated permission from the Regional Ethical Review Board was obtained (Dnr 885–16). Informed consent was secured following both verbal and written information about the nature and purpose of the study, and voluntariness of participation. The raw data (audio files) and the transcripts was stored securely according to standard protocols for sensitive research material and data is presented as to avoid identification of the participants. The raw data (audio recordings) and the transcripts are stored according to legally mandated security protocols, and are accessible only through specific permission from the Swedish Ethical Review Authority.

## Results

**Fig 2:**
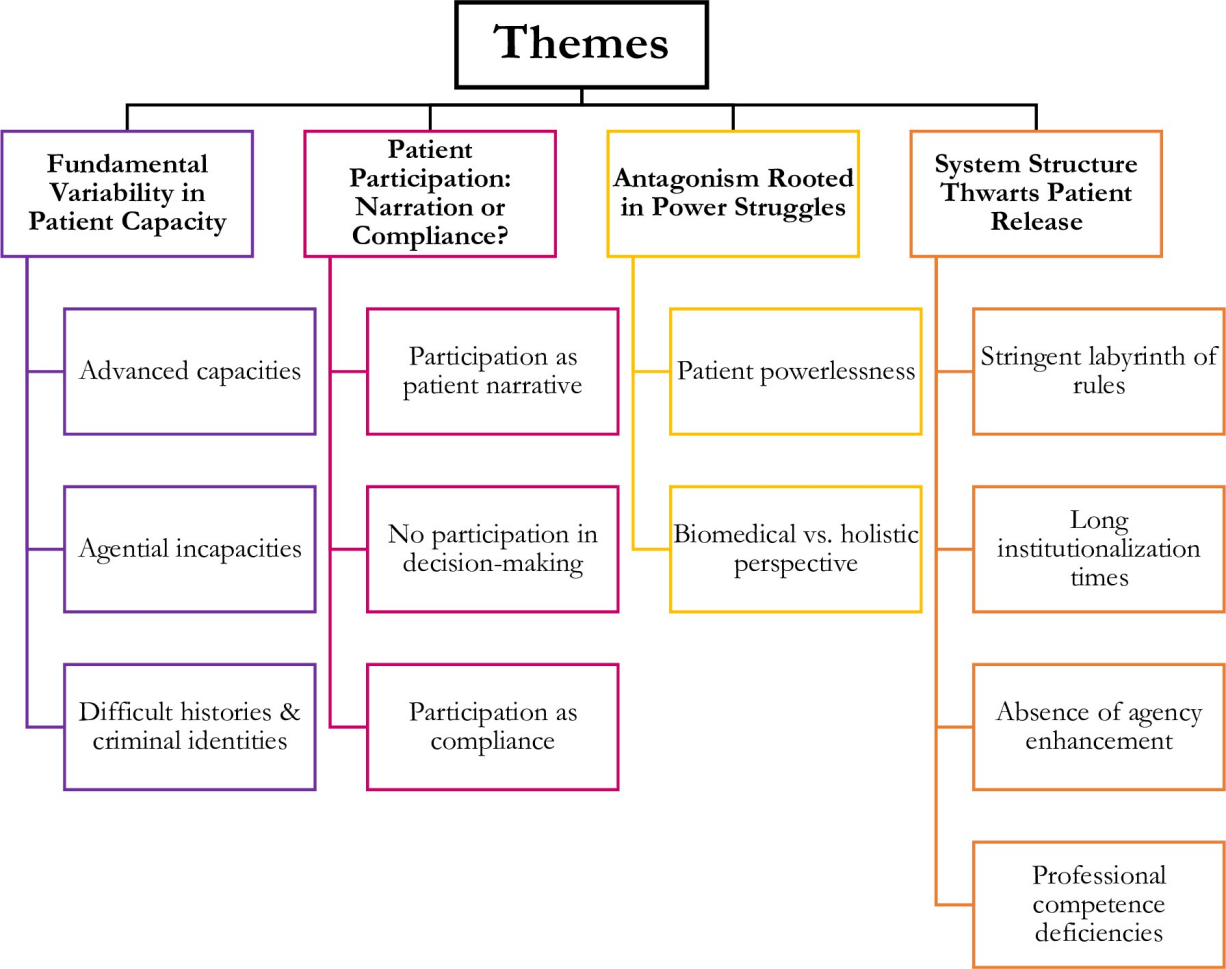
A tree diagram representing the four themes and their subthemes.

Four complex themes emerged from the data “Fundamental Variability in Patient Capacity”, “Patient Participation: Narration or Compliance?”, “Antagonism Rooted in Power Struggles”, and “System Structure Thwarts Patient Release”. Each theme included two to four subthemes (please see Fig 2).

### Theme 1. Fundamental variability in patient capacity

All participants underlined that different patients possess substantially different agential capacities, and that some have difficult personal histories and/or developed criminal identities. Some patients were said to have generally advanced agential capacities, while others were described as suffering from general or particular incapacities. Participants also described patients as having varying thresholds to motivation, as well as different interests, intelligence levels, and tendencies for moral reflection or responsible decision-making.

#### Subtheme 1a. Advanced capacities

Some patients were said to be quite capable of making decisions and standing by them, taking responsibility, moral reasoning about rightness and wrongness of own actions, self-reflection and being aware of own wrongdoings, exercising adequate control over actions, feeling regret over past crimes, as well as relating to others.

I still think they often have, or most patients, they have the ability to talk about, or reflect upon, moral aspects of their actions, too. *(Participant 2)*

Because I think of all the setbacks…emotional explosions that they go through all day, […] wrong medication, emotions that they try to process simultaneously as a group, with coercion, I think they have better self-control than we do. *(Participant 3)*

You have many who have had this self reflection and gone through…been seeing and thinking and looked at it from all angles, that ‘I wasn’t well then, when I did it, poor old people sitting there, they must have been scared senseless, I understand that the police weren’t happy with me either’. They see every aspect… *(Participant 4)*

We have patients who are very high-functioning and with them you can have very constructive conversations and they can have a great deal of understanding for other people. *(Participant 6)*

In this respect, some participants provided examples of patient actions revealing advanced cognitive processes and abilities. One example was that of patients having discussions with caregivers or among themselves about institutional rules, justice, as well as their own behavior in a moral light. Other descriptions highlighted some patients’ ability to establish and maintain business-like arrangements with each other on the ward.

They are very well aware of the rules, and they can relate to them in adequate ways. Sometimes they may question the unit’s rules, guidelines, ‘what’s the point of this?’, they want, they, usually they just want a satisfactory explanation to why we do something in a certain way or why. *(Participant 1)*

And we can clearly tell, because they do business with each other, and we try to…We change their rooms quite often to break up the constellations they create. And many are, I almost called it businessmen, but I mean they trade and sell and buy. And two of those can’t be kept together, because they have some trade in the making. *(Participant 8)*

#### Subtheme 1b. Agential incapacities

Certain patients were described as having poor insight into their conditions, serious intellectual impairment, and/or low cognitive function; all of which were perceived to be fundamental barriers to understanding certain care aspects, making decisions, and participating in care. Other patients were said to struggle with impulse control, addiction to drugs, rational decision-making, and taking responsibility; as evident by being short-sighted, inaccurately assessing own abilities, setting unrealistic goals, making excuses for wrongdoing, or blaming others for own mistakes.

They may have a low intelligence level. So some of them may not be able to understand contexts or make very good decisions. *(Participant 1)*

It can, the answer may be that the patient is too intellectually impaired, we can discuss strategies but when the patient is involved in it they don’t have the tools to actually use the strategies we discuss. […] So that is another large obstacle that…that they don’t have the capacity to use it either. *(Participant 8)*

And it can be a thing like that, that they can’t really evaluate their abilities and they really want to, they can say things like ‘yes, I want to start working right away and I want to work full time’, without actually understanding what that would mean. *(Participant 5)*

#### Subtheme 1c. Difficult histories and disordered identities

Participants described how difficult personal histories of mental illness, crime, drug abuse, and lack of fundamental education or work experience tended to weaken some patients’ agential capacities, or undermine the room for discharging them effectively. Some patients’ criminal identities, whether adopted by the patients themselves or due to their negative portrayal in society outside the institution, were also seen to impede both the discharge of agential capacities (moral reasoning, decision-making, action, responsibility) required for care progress and the practical chance of re-entering society.

And then, most have lived a life shaped by crime, drugs, misery… It’s the kind of life they’re used to, it’s all they know. *(Participant 1)*

We have patients who have never worked a day in their life. *(Participant 4)*

[…] if you don’t have that from school and all, which many of our patients don’t, I mean, they don’t have that level of education. Then you cannot process information in the same way, or understand. It’s like… It’s fundamental, having gone through school and preferably high school and such, and maybe had a few jobs, then you know and are able to follow the world around you. But if you dropped out in ninth grade and have been doing drugs since then… *(Participant 6)*

But there are patients who have committed very grave crimes, and if somebody in the street were to hear about those crimes they would probably say that they should be locked up for the rest of their lives because they’re so dangerous. *(Participant 2)*

### Theme 2. Patient participation: Narration or compliance?

When participants spoke of patient *participation* in the care processes, no unified definition was provided but instead different implicit ideas revealed themselves. Which notion of participation was implicitly assumed by participants also colored their views regarding the opportunity for patient participation. Caregivers who assumed that listening to patient narratives (including wishes or opinions) is sufficient for patient participation, also perceived the room for such participation to be larger. Others who understood participation as involving more than simply listening to patient narratives, perceived the room for patient participation as illusory or absent. At the same time, there was a general tendency to equate patient participation in the care with compliance to clinical decisions and institutional rules. The latter was often used to assess patients’ progress in the care and was sometimes incorporated into patterns of rewards and punishment to influence said progress.

#### Subtheme 2a. Participation as patient narrative

In some cases, participation was understood as providing patients with opportunities to be heard through stating opinions, preferences, or wishes. The availability of such opportunities were seen to be indicative of patient influence over care decisions.

I would say they have every chance in the world to participate. And influence. On every patient team meeting, every six weeks, they get to evaluate how the past six weeks have gone and what they have thought about activities and so on, do they want to continue, do they want to stop, do they want more, do they want to change completely… So they have that chance there, to share their opinions with everyone in the team. *(Participant 4)*

Right now I would say at our unit that we are very good at listening to what [the patients] want. For example, some want to do a certain type of activities, others want to do other… I think it’s good also that the patients can take some advice or… If you put it like this, ‘have you considered doing this instead?’ *(Participant 7)*

#### Subtheme 2b. No participation in decision-making

Other participants explicitly stated that patients do not *truly* participate in the care, in the sense that what patients say does not affect the decisions being made, as they are not part of the decision-making process.

[…] on one hand we do, as I said, have weekly meetings and talk to them. On the other hand they don’t participate in the actual decision-making, even though they give their opinions, which is actually quite different. *(Participant 2)*

Well, a patient who participates and takes part in their patient team in the way it’s planned, meeting their contact person, preparing, checking their treatment plan, participating in that and bringing it to the team when meeting the others—such a patient is involved to, what should we call it, ninety-eight percent, they have insight and participates. But they still have very little to do with the decision-making […] and with the structure that we have here, it’s hard to say that the patient participates. *(Participant 3)*

Despite some participants stressing an ideal of honest and open communication with patients, they also stated that this is not the practice. The lack of transparency about care decisions and keeping patients in the dark about certain aspects of their own situation were deemed problematic.

Sure, there can be times when decisions…are made behind their backs, but usually we try to be as open as possible. *(Participant 1)*

Many patients frequently bring up this: “I spoke to a doctor the other day. Why does he say that when we talk, and then we meet in court and then he says this? That was completely new to me. And then came an expert who doesn’t even know me, and now I got this sentence […] and it’s your fault because you wrote that in my records and I haven’t been offered to read that.” So I think that a lot of this that I keep coming back to, transparency, is a question of not just having it one day a year. You cannot have it the day they get their sentence, or the day they’re going to the court of administrative law. I think we need to try to keep it all the time. *(Participant 3)*

#### Subtheme 2c. Participation as compliance

Many participants perceived the value of patient participation as linked to securing patients’ compliance to care decisions and institutional rules. These participants also perceived such compliance as a sign of successful patient participation, or in effect, equal to participation, regardless of whether patients agreed to the rules or care plans. Similarly, patients’ refusal to comply was seen as equivalent to poor participation in the care.

If you’d had a better alliance with certain patients and got them with you, so to say, that we agree that this is what we need to do, then rehabilitation could have gone faster in many cases. When they oppose the treatment, refuse to accommodate when it comes to medication, maybe try to hide things away…We have agreed on activities that they need to do, to activate themselves, and they cancel, don’t show up, run away when on leave, then they constantly take steps backwards in the process and have to start over… so…Yes, it depends, depends a lot on their own will and, and on participation. *(Participant 1)*

They can participate very actively if they share our ideas. It’s more, they usually don’t. And then, I mean, they can participate and say ‘I’m not going to take this medicine’, and they don’t have to do that, but then they don’t get out either. So, I mean, they can participate that way too, the negative way. But there is not much happening for them if they don’t work with us. So it, but it’s our job to make, get them to join us. *(Participant 6)*

Compliance, especially without complaints or objections, was also described to be a core measure of care progress and a condition for reward, in the form of parole or permanent release, or punishment through maintained or tightened restrictions. The compliance view of participation took precedence over all other considerations such as moral reasoning, shared decision-making, and opportunity for taking responsibility, which could otherwise be seen as ingredients in participation.

Those who have seemed, well, relatively pleased, those who haven’t caused much fuss and conflicts, those who take care of themselves very well, those have…they get somewhere. They get released. *(Participant 8)*

We’ve had patients who don’t want to work with us when it comes to medication, and refuse to take their medicine. And then they don’t, but then they will remain at the unit in an emergency room until they start to reconsider or until we can convince them. *(Participant 6)*

And then of course it’s not just about whether they can make moral decisions, but…as long as they’ve behaved in compliance with certain points. *(Participant 1)*

I will still come here tomorrow and get paid for being here at work tomorrow. You will be here one day longer. Because you’re not participating in your treatment. *(Participant 4)*

### Theme 3. Antagonism rooted in power struggles

Participants described the power structure of the care as a strict hierarchy, with doctors on top and patients on the bottom. Many participants stressed how decision-making power is unevenly distributed among professional roles, with physicians getting the lion’s share of responsibility for principled high-impact decisions. Several participants highlighted an institutionalized decrease of decision-making prerogative, authority, and custodianship between professional groups, as well as between professionals and patients. Although physicians, nurses, care assistants, and orderlies are all part of the team, their decision-making power is stepwise limited in this order. Patients reside in the bottom of this hierarchy, having no formal decision-making power, very restricted freedom, and no control over how the care proceeds. This was said to lead to patients feeling powerless and rebelling against the system, which in turn, creates major conflicts and disagreements between patients and their caregivers. Another level of antagonism was illustrated as a tension of values and outlook on patient care between physicians and caregivers. Participants described the hierarchal structure to limit their own decision-making power and latitude to adapt the care to the individual patient and situation. A particular objection voiced in this regard involved a strong disagreement with physicians’ excessive focus on medicating patients and their lack of a holistic perspective on patients.

There is a very clear structure and hierarchy here, and I can feel that it trickles down through the categories of professions too, down to the patient. *(Participant 3)*

#### Subtheme 3a. Patient powerlessness

With patients being at the bottom of a visible power hierarchy, the participants often described them as feeling disadvantaged, powerless, and helpless. As a result, some patients were said to develop very negative attitudes towards the staff and institution or even reject the forensic psychiatric system in its entirety.

Even though you don’t want this ‘us against them’ meaning patient against staff, it happens automatically because we have the keys and the keycards, and we can move freely while they cannot. *(Participant 4)*

And then the patient will of course feel that they don’t get what they need, and that they’re not being listened to, and that we don’t understand that they know more about themselves than we do, and thus feel powerless. *(Participant 3)*

[…] some patients think prison would have been better, knowing how long they were in for, it’s a specified punishment. So yes, some patients are against the whole system. All of forensic psychiatry. *(Participant 2)*

The perceived powerlessness of patients was illustrated in the context of a pervasive threat of force and coercion against them. Decisions involving coercion and use of force against patients were described by the caregivers as difficult to make and ethically problematic, despite being sometimes motivated by care routines or necessary for safety reasons.

The patient knows that if “I don’t do this then I will be restrained or get a forced injection”. *(Participant 3)*

We try to prevent [using force] very much […] to prevent situations with restraining or forced injections and such. But when it happens it’s always tough. It always gets to you. *(Participant 8)*

I think there are always ethical problems when there is force involved, for example, if you restrain a patient…It’s always difficult, when should you do it and when should you not, what is best for the patient…So there is always a, I think, a dilemma. *(Participant 2)*

I mean, if a patient gets manic […] then we will eventually have to force-medicate, otherwise they will burn out their brain, and then, I mean, we don’t want them to get that kind of damage. *(Participant 6)*

Caregivers also linked feeling powerless to patients seeking other ways to gain power inside the coercive and restrictive system, either through escalated dissent which leads to confrontation or through attempts to manipulate caregivers for parole benefits or access to desired medications. Some participants reflected that such negative patient feelings and their antagonistic relationship to caregivers could contribute to adverse clinical outcomes such as relapse to drug abuse, recidivism, or breaching rules; all of which undermine the goals of care and frustrate care progress.

Then it can also be things like doing it as an act of protest, because they’re displeased with their situation or something else that has been resolved. *(Participant 1)*

[…] right now we have many patients who strike because they don’t get higher doses of their medication, and “then I won’t do shit, I’m not going to move a finger”. (*Participant 4)*

So some are probably, they behave and say that they accept responsibility because they, it looks and sounds good. And I think it’s hard to determine what is what really…*(Participant 2)*

And it’s like daily agony, or if there is anything else, in their personal lives or on the outside, that things happen and they may be a bit…emotional one day, and a fellow patient comes in with drugs, and then it’s hard to say no, even if they really want to. *(Participant 8)*

#### Subtheme 3b. Biomedical vs. holistic perspective

Participants highlighted a general absence of holistic perspective, and a tension between how they believe patient treatment should proceed and how it does in reality. The physicians’ narrow focus on (over)medicating patients was reported to be morally problematic, particularly in cases where patients struggle with drug addiction, as overmedication potentially worsens their addiction. Some participants also believed medical management of patients to be inadequate with presence of gaps in treatment including a lack of active addiction treatment as well as insufficient focus on psychotherapy.

Well, I don’t know, we, we don’t really actively do much to treat addiction, but we gather addicts, we give them medicine…*(Participant 8)*

I don’t believe that an active addiction can be medicated away with other substances. First of all we need to work on making the person aware that they have an addiction, and making them want to get rid of that abuse. And if they don’t want that, then they can claim that they have all sorts of diagnoses and problems and needs to get that addiction. And we have no way of working with addiction within coercive psychiatric care if we don’t choose to be a part of that work. *(Participant 3)*

For my part, the ethical thing would be that they are being medicated in ways that I am not entirely comfortable with. In many cases. *(Participant 4)*

I mean, we start at the wrong end. We medicate first, and then there is the discussion “yes, but perhaps we should start a psychological evaluation or something”. *(Participant 8)*

### Theme 4. System structure thwarts patient release

The participants described FP care system including the coercive nature of its daily care, strictly regulated processes leading from court-ordered admission to conditional gradually relaxed restrictions, regular court hearings every six months, paroles, outpatient care, and final discharge, all of which comprise multiple layers of complex interaction. When these layers are put together, they bring about a system which structure seems to lead patients to continuously fail at (permanently) escaping it. This is due to the combination of rigid and sometimes contradictory rules which are difficult to navigate, unnecessarily long institutionalization times, little focus on training and improving patient capacities leading to their dependence on the system, as well as deficiencies in caregivers’ training and competence engendering non-standardized evaluation of patients and their progress.

One form of coercion is to refer the patient. When the patient is referred it’s not actually a friendly referral where they have a choice, but in this coercive care the referral is really one step before taking other coercive measures. *(Participant 3)*

[…] it’s a bit of a Catch 22 situation with many of our patients […] Yes, but you can get out of here if you participate actively in your own treatment plan. Which is a very difficult thing for many to realize. *(Participant 4)*

#### Subtheme 4a. Stringent labyrinth of rules

The structure of the FP care system was described by most participants as coercive, restrictive, and rigid to both patients and caring staff, mostly based on the prioritization of safety requirements for the surrounding society. The stringency of the system was sometimes viewed as unnecessary or as an obstacle to patient treatment. Institutional rules were also described as contradictory and thus confusing to patients. Nonetheless, some participants found that the restrictions are justified or that more invasive, albeit ethically problematic, measures might sometimes be needed.

I have to follow the rules as much as the patient does. *(Participant 4)*

[…] and at some point with all the coercive measures there are just too many stops and obstacles along the way and you end up forgetting why, why did we take these measures and how do we go back, and can we really let go now that this happened. And it gets. it’s the compulsory care in itself and there are so many things to consider, security and such. That sometimes makes it stand still, I think. *(Participant 3)*

We have very high security that may not be necessary for our patients, but can instead be negative or take away a lot of the motivation. *(Participant 6)*

[…] it gets too vague and fuzzy for them, they have expressed that themselves and I agree with them. That it gets confusing when one time we’re ruled by laws and regulations, but then all of a sudden we’re supposed to do it like this, as… where is the security? For example, now we’re going to stand in a kitchen with knives and things like that, because we’re supposed to cook together. *(Participant 4)*

And of course, it’s an extreme violation to perform such a [body cavity] search, so at the same time I understand, but sometimes you can when you notice, and you hear that things are circulating at the unit, drugs and other things, you sometimes wish for the sake of security that you could do more. *(Participant 1)*

#### Subtheme 4b. Long institutionalization times

The strict system was stated to lead to extreme and unnecessarily long institutionalization times, which itself was seen to contribute to frustration and hopelessness for both patients and staff. Particular sources for the long institutionalization, as explained by the participants, involved a disproportion between patient non-compliance and routine punitive consequences in the form of tightened restrictions that set back the care process to earlier stages, as well as an bottleneck problem external to the institution due to support shortage for discharged patients or those transferred to outpatient care. This was seen to negatively impact patients’ acceptance of the system and their motivation to achieve progress in the care.

It is, I generally think that the treatment time-spans are a bit extreme, a bit too long, since they are generally here for about five and a half years. And many patients could be released sooner. *(Participant 6)*

And it can be quite hard when you meet a, a patient who has been in psychiatric treatment for thirty years. And a young [caregiver] as they sometimes say, lectures them or comes up with a suggestion that they might have tried already twenty years ago and it didn’t work. Then it can be hard to motivate that “hey, let’s try again”. *(Participant 8)*

Then there are patients who may have a point, that they have committed a very, not a serious crime actually, but after they were convicted they’ve been unruly so they’ve been kept for a longer time. For example if they just shoplifted or something, and then they stay here for five years because they start fights and spend all their time at the clinic. And then they can think “but why am I here still after five years, I just stole a thing” […] They get angry with us because they’re frustrated with life. *(Participant 2)*

One can feel very limited in some situations. And because, knowing that we have done all we can for this patient, and the patient is more or less done with treatment here. But we’re going round in circles because the other participants have no room and the patient cannot be taken in for non-institutional care and so on. […] We can be very limited in that situation because we have no power over non-institutional psychiatry. And their staffing situation. *(Participant 4)*

Sometimes we have to start over with patients who were actually on their way of being released, during the time they were waiting for housing, because of the dynamics at the unit or drugs coming in that actually ruin the environment. *(Participant 3)*

#### Subtheme 4c. Absence of agency enhancement

In addition to the long institutionalization times, participants also lifted a lack in the care to enhance agential capacities, needed for life outside of the care facility, as a contributing factor to patients’ dependence on the care system. Although participants stated that they saw value in responsibility training and in having discussions about crime, they also said that no emphasis was placed in the care on training patients to become (more) independent or responsible, or on helping them process their crimes. Some patients were said to develop far-reaching dependence on the daily institutional routines and the assistance provided by staff. Patients were described as becoming too dependent on- or comfortable within- the system, such that they either do not want to leave or have a desire to come back after being released.

[…] I think this is something we could probably work on more, making them take real responsibility *(Participant 2)*

In some cases I think we could focus more on how [patients] reason and so on. *(Participant 1)*

And I believe the staff members probably don’t bring [patients’ crimes] up often enough. So perhaps we should talk about it often, because [patients] do…They think about such things a lot. *(Participant 2)*

We have patients who don’t even…who say no to several housing offers because they feel so comfortable at the unit. […] I find it quite tragic that someone settles with that and even strives to come back, and wants to come here. *(Participant 5)*

We are the patient’s comfort blanket, so when they leave us they bring the comforter with them. We don’t just pull it away, because many of them need it for some more time. We have always said that, that they can just call us if they want. *(Participant 4)*

#### Subtheme 4d. Professional competence deficiencies

At the same time, participants questioned the adequacy of their own qualifications and training and whether they make possible constructive adjustment in the daily care and provide standardized patient evaluations. The latter idea also lead some participants to question whether patient outcomes and decisions relating to the restriction of their freedom could change with different staff dynamics.

I mean we’re not trained in specifically treating addiction or having that type of conversations. *(Participant 8)*

I mean in general you don’t have the knowledge and know very little about psychiatry. *(Participant 6)*

But, but it gets more difficult for staff members, seeing to those aspects [patient’s reasoning], because it’s very individual. ‘I think this is okay, in my opinion this is good’. While others would say ‘no, but I don’t think this is okay’. *(Participant 1)*

I mean, of course, since treatment is given by humans and we use some type of evaluations that are subjective, and we have ourselves to relate to when doing our job. So I think it depends on the time of the day, what part of the workforce is on duty, it will of course look different. And that’s worth thinking about, is this something we do because we feel unsafe in the group or could we have handled this differently if we had been a different constellation of colleagues. Is this patient getting this restriction or these reprisals because we cannot handle the situation under these circumstances? *(Participant 3)*

## Discussion

The results of this study should be read against the backdrop of the basic conception of PCC mentioned at the beginning of this paper: a holistic view of patients, recognition of a broad patient narrative, and continuous SDM based on these. Such PCC strives for- or assumes- an alliance between patients and HCPs, with goals to emancipate patients, promote adherence to care plans, increase patient satisfaction, and improve resulting health outcomes. Finally, SDM comes in different variants of which only the ones implying high-level dynamics, with patients having control over the goals and conditions of the care, are uniquely set to promote a strong emancipation goal [15]. Nevertheless, less advanced SDM variants may still give room to promote other goals of PCC.

Most patients were described by the participants to possess agential capacities necessary for PCC, albeit these capacities were also seen as fragile, easily undermined, and difficult to maintain or enhance. A smaller group of patients were held out as lacking fundamental agential capacity due to chronic intellectual or cognitive limitations. In this latter group, one could see a room for listening to patient narrative but a very limited room for SDM. Therefore, while decisions taken by clinical teams may still take into account what the patient says, there might be no room for patient empowerment or influence as well as little room for continuity or HCP-patient treatment alliance, due to patients’ challenged comprehension, memory, or functionality. Thus, the aim pursued in this limited kind of patient participation would be confined to maintaining security on the ward and adherence to care decisions and plans, such as prescribed medication and activities. The long-term chances of care progress for this group was painted by the participants in a bleak light, with long institutionalization times and psychological institutionalization effects as main hurdles. It is difficult to envision how more advanced PCC-strategies could overcome such hurdles for this patient group.

At the same time, we do see a room for a type of personalized FP care, where patient narrative and less advanced variants of SDM as described by Sandman and Munthe [15] are used instrumentally. This kind of individualized care would then be employed to maintain security, effect adherence, and to some extent, achieve some degree of patient emancipation albeit with security prerogatives of the surrounding society setting limits to the scope of adjustments that HCPs can make. This possibility is consistent with the conclusions of Allerby et al. [27], whose study revealed that staff do not perceive their patient’s psychotic symptoms or involuntary setting to be a hurdle for PCC implementation.

For the more capable patient group, PCC or patient participation involved different barriers, viz. the fragility of these capacities, the antagonism between patient and FP care, and an institutional structure rigged for failure. There is clearly no way around the basic conflict between a patient’s desire to be free and rule their own life, and the societal norms and values that motivate FP care in the first place. The latter’s inability to assume the sort of therapeutic alliance taken for granted in most other healthcare contexts, constitutes a fundamental challenge to the idea of person-centered FP care. However, we believe that the results still describe a limited but important room for a particular variant of PCC that also includes some high level SDM dynamics.

Sandman and Munthe [15] describe three (out of a total of nine) high level dynamic versions of SDM, of which two are incompatible with the conditions of FP care, even when patients possess basic or advanced agential capacity. These two levels imply either that patients are free to do whatever they please regarding the care even if HCPs disagree, or that HCPs may adapt to whatever the patient desires in order to achieve a joint decision. However, the SDM variant called *Professionally Driven Best Interest Compromise (PDBIC)*, seems to allow for a situation of basic therapeutic antagonism and impossibility of full emancipation. PDBIC does this by having HCPs openly accept strategic compromises in cases of disagreements with patients, in order to establish a therapeutic alliance, promote adherence, and increase treatment success.

The openness of such strategic compromises in turn promotes a minimal level of empowerment for the patients, as they are given a choice on how to react to the HCP’s strategy. In standard healthcare settings, the disagreements calling for such compromise may view established clinical standards, resource availability, ethical considerations in terms of acceptable risk exposure of patients, distributive justice concerns, and others. In FP care, these elements include legal requirements as well as norms about ward security and public safety. This implies that the available *windows of compromise*, i.e., the range of possible trade-offs or solutions to conflicts of interest within FP, might be further (or at least differently) restricted. Such overlapping roles and responsibilities of professional caregivers often leads to moral stress or distress [28,29] and can adversely affect ward atmosphere [30]. This is respectively reflected in participants’ reflections on the difficulty of using force with patients (under subtheme 3a) as well as their focus on patients’ compliance and importance of security measures.

Yet within the space of such *windows of compromise*, caregivers may coax patients into, at least, instrumentally accepting the FP care goals, and better managing the many barriers to treatment success as described in the results section. However, while there is a theoretical room for FP caregivers to make continuous PDBIC adjustments to the care, the extent to which said adjustments are compatible with the goals of care requires further analysis. First, the non-negotiable restriction to compromises has to be identified in order to carve out the potential *window of compromise* and ensure transparency with patients regarding the room for influencing decisions. Second, there is a need to identify, more specifically, the unnecessary restrictions or structural obstacles which could be open to discussion or simply in need change. Third, since PDBIC implies the use of open strategic action to influence patients’ acceptance of care ideals, it needs to be clarified how well caregivers are able to distinguish between necessary restrictions and care ideals, and still be willing to accept a compromise within the explicit restrictions.

Notably in the results, the *windows of compromise* might be different depending on the professional role. The respondents in this study (not being forensic psychiatrists) described themselves as also being restricted by decisions made higher up in the hierarchy, and one could explore whether a modified version of the PDBIC need also be applied to the interaction between caregivers and senior psychiatric consultants. Examples given by the participants regarding the mechanisms behind undesirable institutionalization and very long treatment times, also pinpoint the importance of how court decisions are made while being advised by senior FP consultants.

Given the results regarding variability of agential capacities and care progress, the nature of FP care, and patient characteristics; the *window of compromise* is also likely to be context-dependent and variable over time for individual patients, situations, and actions. Hence, the variability in patient agential characteristics is not problematic but rather supportive of the individually adapted approach that PDBIC offers. The problem lies in establishing the *window of compromise* for each patient and adapting this consistently, even with different caregivers.

The conflicting views from the participants regarding the actual presence of- and conditions for- patient participation in the care, as well as the tendency to equate participation and compliance, is consistent with what little research has been done in this area [9,10]. But it also highlights a challenge for the caregivers to distinguish in practice between necessary restrictions, caring ideals, and instrumentally motivated actions which are open to adjustment in cases of disagreement with patients. Hence, the room for coercion, dissent, and disagreement should be part of the conceptualization of what *window of compromise* is available in a particular instance. This may require extra specialized training for FP caregivers, even if they may possess general professional competence, as the ability to handle conflict without confrontation or hostility is among crucial nursing competencies in FP, for instance [31,32].

We see two main challenges for implementing the PDBIC version of PCC in FP. The first challenge links to the fact that PDBIC rests on an assumption of transparency towards patients about the *window for compromise* and the various conditions surrounding it. Otherwise, the strategic adaptation of care routines to patient desires, in order to promote adherence and treatment progress, cannot be openly made in a way that empowers patients. Here, the results regarding contradictory rules, lack of a holistic approach, and non-standardized evaluations of patients constitute obstacles to this approach that might need both cultural and institutional reform to be overcome. This requires further analysis to clarify to what extent such reform is consistent with the basic legislation in different jurisdictions.

Second, the apparently counterproductive Catch-22-like mechanisms, i.e., when attempts to overcome a certain impediment fail due to contradictory requirements of said impediment, described in the fourth theme stand out as a major barrier to having SDM á la PDBIC to improve care outcomes, patient adherence, or patient satisfaction. Indefinite patient release is thwarted within the system because of a vicious cycle of patient powerlessness (from the third theme), non-compliant behavior, and prolonged institutionalization. In a system where patients are stripped of power, the one way to gain a semblance of control is to do exactly opposite to what the rules dictate: dissent. Because a lack of compliance is punished by going back to square one, patients who strike or act in rebellion end up staying longer inside the system, which in turn leads to more frustration and loss of hope for both patients and staff. This may lead to patients engaging in further destructive behavior, like drug abuse, which again feeds into their non-compliance to rules and leads to further cycles of punishment, prolonged institutionalization times, frustration, disempowerment, and so on. Once one adds a lack of opportunities for responsibility [33] and agential capacity training, it becomes clear how this links to patient’s dependency on the system whereby even patients who are released end up being readmitted. This phenomenon is known as the revolving door [34,35], and creates tension between a patient’s desire to be free, which itself is a main source of staff-patient disagreement, and an urge to stay within the safety of the institution.

In order to avoid the apparent counterproductive mechanism of the system, there has to be a room for trial and error, or even failure, as well as a space for disagreement. If SDM will be used as a training tool for improving agential capacities we also have to accept that, once capacities become sufficiently advanced, patients might question rules and disagree with them. Therefore, allowing for conflict becomes essential. However, if we are to conceive of PCC in FP, we must accept a PCC version where the emancipation aim is constricted and the action level of SDM is not practiced as a full-blown high-level dynamics beyond strategic compromises limited by external rules. Rather, the PDBIC variant of SDM would be used as an instrumental tool to develop and maintain agential capacities and (at least, conditional) acceptance of the externally defined care goals in terms of ward security and public safety. The rigid system provides no room to foster responsibility in this manner and creates an incentive for patients *not* to admit responsibility. This makes it practically impossible to promote PCC as an agency-mobilizing healthcare provision model, since patients and staff are not on equal footing in the hierarchal power structure of FP care.

## Conclusion

FP caregivers with daily patient contact paint a partly contradictory, partly bleak image of the room for PCC or related patient participation in the Swedish context. While only a minority of patients are seen as incapable to participate (due to cognitive limitations), fragile and variable agential capacities, a basically antagonistic relationship between patients and caregivers (and the care institution), and complex institutional barriers clearly constrain the room for PCC, albeit participation strategies may still be used instrumentally to influence patients’ adherence. Having said that, the analysis in this study might have been limited by the caregivers’ point of view and might have been enriched further through the addition of psychiatrists’ or patients’ perspectives. However, this was not possible at the time of data collection and will need to be pursued in other projects. At the same time, we have described a room for some advanced, yet constrained PCC which employs high level SDM dynamic capable of empowering patients, and how this may be used to harness care strategies to improve treatment adherence and success. Implementing such a practice shift will probably require educational interventions, and both cultural and institutional adjustment and reform.

## Supporting information

S1 Appendix Interview guide, Swedish. (DOCX)

S2 Appendix Interview guide, English. (DOCX)
