# Supplementary material for: Caregiver perspectives on patient capacities and institutional pathways to person centered forensic psychiatric care
Source: PLoS One. 2022 Sep 29;17(9):e0275205. doi: 10.1371/journal.pone.0275205 (PMC9521939; doi:10.1371/journal.pone.0275205)
Supplement: S1 Appendix — (DOCX) [file pone.0275205.s001.docx]

**Appendix 1: Interview guide, Swedish**

Personalens syn på patienters moraliska agentskap och förmåga till ansvar i rättspsykiatrisk vård

Intervju-guide (Utkast)

2016-09-06

1. Berätta om ditt arbete med patienter här på kliniken
   1. Din profession?
   2. Vilken diagnostisk kategori tillhör patienterna du arbetar med?
   3. Hur länge har du arbetat här, tidigare erfarenheter?
   4. Vanliga inslag i vårdarbetet: årgärder, beslut, planering, tvångsinslag
   5. Din egen syn på vad som är målet med den vård du är involverad i
   6. Tycker du att du ofta ser etiska konflikter, vilka är i så fall dessa?
2. Som läget är nu, hur delaktiga skulle du säga att patienterna är i vårdens genomförande och i olika beslut om hur vården ska se ut?
   1. Möjlighet att göra sig hörda med avseende på detta (hur?)
   2. Medverkan i utförande av vissa inslag i vården (egenvård)
   3. Möjlighet att delta i beslutsfattande, utvärdering och/eller planering av vård
   4. Möjlighet att ha inflytande över beslut, utförande, planering, utvärdering, m.m.
   5. Din möjlighet att anpassa beslut, utförande, planering efter sådana individuella variationer.
   6. Finns det inslag i vårdens förutsättningar eller organisation som hindrar en högre grad eller vissa former av delaktighet?
3. Händer det att det är oenighet med patienten om vården och hur hanteras detta?
   1. I vilken sorts situationer? Ofta?
   2. Tar du hänsyn till patientens åsikt eller perspektiv? Hur?
   3. Vem tycker du ska ha det avgörande inflytandet över hur vad som beslutas? Varför?
   4. Vilken handlingsfrihet upplever du dig själv ha i sådana situationer, att anpassa beslut och agerande utefter patientens inställning?
   5. Tycker du alls att det är önskvärt med sådan anpassning - hur och varför?/varför inte?
4. Hur bedömer du dina patienters förmågor till eget *beslutsfattande*?
   1. I allmänhet?
   2. I relation till olika sorters inslag i vården?
   3. Vilka aspekter av beslutsfattande är det du särskilt noterar i relation till dina patienter? (Informationshantering, föreställningar om världen, önskningar/värderingar/mål, känslomässig kontroll, resonemangs- och slutledningsförmåga)
   4. Är vissa patienter mer/mindre beslutsförmögna än andra - vilka/när?
   5. Vad anser du att en patients beslutsförmåga innebär, t ex avseende etiska frågor i vården
   6. Finns det inslag i vårdens förutsättningar eller organisation som du upplever hämmar eller stärker patienternas förmåga till beslutsfattande?
5. Hur bedömer du dina patienters förmåga att *kontrollera sina handlingar och sitt liv* utifrån sina egna beslut?
   1. I allmänhet?
   2. I relation till olika sorters inslag i vården?
   3. Vilka aspekter av kontroll är det du särskilt noterar i relation till dina patienter? (fysisk förmåga, förmåga att behärska känslor och impulser, förmåga att resonera moraliskt)
   4. Är vissa patienter mer/mindre förmögna till kontroll än andra - vilka/när?
   5. Vad anser du att en patients förmåga till självkontroll innebär, t ex avseende etiska frågor i vården.
   6. Finns det inslag i vårdens förutsättningar eller organisation som du upplever hämmar eller stärker patienternas förmåga till självkontroll?
6. Hur bedömer du dina patienters förmåga att *göra moraliska bedömningar*?
   1. I allmänhet
   2. Av beskrivna situationer som inte innefattar dem själva
   3. Av sina egna handlingar (tänkta eller verkliga)
   4. Av dina och andra vårdprofessionellas handlingar
   5. Av den rättspsykiatriska vårdens utformning och organisation
   6. Vad anser du att en patients förmåga att göra moraliska bedömningar innebär, t ex avseende etiska frågor i vården
   7. Finns det inslag i vårdens förutsättningar eller organisation som du upplever hämmar eller stärker patienternas förmåga till moraliska bedömningar?
7. Hur bedömer du dina patienters *förmåga till ansvarstagande*?
   1. I allmänhet
   2. I relation till att medverka i olika inslag i vården
   3. I förhållande till den lagöverträdelse de är dömda för
   4. I relation till åtaganden och överenskommelser som du gör med patienterna
   5. Vad anser du att en patients förmåga till ansvarstagande innebär, t ex avseende etiska frågor i vården
   6. Finns det inslag i vårdens förutsättningar eller organisation som du upplever hämmar eller stärker patienternas förmåga till ansvarstagande?
