# Supplementary material for: Caregiver perspectives on patient capacities and institutional pathways to person centered forensic psychiatric care
Source: PLoS One. 2022 Sep 29;17(9):e0275205. doi: 10.1371/journal.pone.0275205 (PMC9521939; doi:10.1371/journal.pone.0275205)
Supplement: S2 Appendix — (DOCX) [file pone.0275205.s002.docx]

**Appendix 2: Interview guide, English**

Personnel view of patients' moral agency and ability to account for legal psychiatric care

Interview Guide (Draft)

2016-09-06

1. Tell us about your work with patients here at the clinic
2. Your profession?
3. What diagnostic category belongs to the patients you work with?
4. How long have you worked here, past experiences?
5. Common elements in the care work: measures, decisions, planning, compulsion
6. Your own view of what is the goal of the care you are involved in
7. Do you think you often see ethical conflicts, which ones are these?
8. As the situation is now, how active would you say that patients are in care implementation and in different decisions about how care should look?
9. Ability to be heard regarding this (how?)
10. Participation in the performance of certain aspects of care (self-care)
11. Ability to participate in decision making, evaluation and / or planning of care
12. Possibility of influence over decisions, execution, planning, evaluation, etc.
13. Your ability to adapt decisions, execution, planning for such individual variations.
14. Is there any element in the care conditions or organization that prevents a higher degree or certain forms of participation?
15. Does it happen that there is disagreement with the patient about care and how is it handled?
16. In what kind of situations? Often?
17. Do you take into account the patient's opinion or perspective? How?
18. Who do you think you should have the decisive influence on what is being decided? Why?
19. What freedom of action do you experience yourself in such situations, adapting decisions and actions according to the patient's attitude?
20. Do you at all think that it is desirable with such adaptation - how and why? / Why not?
21. How do you assess your patients' abilities for their own *decision making*?
    1. In general?
    2. In relation to different types of care elements?
    3. What aspects of decision making are you particularly aware of in relation to your patients? (Information management, world-wide views, wishes / values ​​/ goals, emotional control, reasoning and end-of-life skills)
    4. Are some patients more / less decision-making than others - who / when?
    5. What do you think the patient's decision-making capacity means, for example, ethical issues in health care
    6. Is there any element in the care conditions or organization that you experience inhibits or strengthens the patient's ability to take decisions?
22. How do you assess your patients' ability to *control their actions* *and their lives* based on their own decisions?
    1. In general?
    2. In relation to different types of care elements?
    3. What aspects of control are you particularly noticing in relation to your patients? (Physical ability, ability to master emotions and impulses, ability to reason morally)
    4. Are some patients more / less able to control than others - who / when?
    5. What do you consider to be a patient's ability to self-control, for example regarding ethical issues in health care.
    6. Is there any element in the care conditions or organization that you experience inhibits or strengthens the patient's ability to self-control?
23. How do you assess your patients' ability to *make moral assessments*?
    1. In general
    2. Of described situations that do not include themselves
    3. Of their own actions (imagined or real)
    4. Of your and other healthcare professionals' actions
    5. Of the legal psychiatric care design and organization
    6. What do you consider that a patient's ability to make moral assessments means, for example, ethical issues in health care
    7. Is there any element in the care conditions or organization that you experience inhibits or strengthens patients' ability to moral assessments?
24. How do you judge your patients' ability to *take responsibility*?
    1. In general
    2. In relation to participating in various aspects of care
    3. In relation to the offense they are convicted of
    4. In relation to the commitments and agreements you make with the patients
    5. What do you consider a patient's ability to accountability, for example regarding ethical issues in health care
    6. Is there any element in the healthcare conditions or organization that you experience inhibits or strengthens the patient's ability to take responsibility?
